# Correction Method for the Bending Characteristics of Aero Ball Joints under High Temperature and High Pressure

**DOI:** 10.3390/ma15134414

**Published:** 2022-06-22

**Authors:** Jie Yuan, Kaijie Yang, Hong Shi, Boxiao Yang, Jiangong Wang, Yanlong Jiang

**Affiliations:** 1 Key Laboratory of Aircraft Environment Control and Life Support, MIIT, Nanjing University of Aeronautics and Astronautics, Nanjing 210016, China; jieyuannuaa@foxmail.com (J.Y.); tiehanhan@nuaa.edu.cn (K.Y.); jiang-yanlong@nuaa.edu.cn (Y.J.); 2 School of Energy and Power, Jiangsu University of Science and Technology, Zhenjiang 212100, China; 3 AVIC Taiyuan Aero Instrument Co., Ltd., Taiyuan 030006, China; yangboxiao1991@163.com (B.Y.); 13633414203@139.com (J.W.)

**Keywords:** ball joint, bending friction torque, bending stiffness, BP neural network

## Abstract

The aero ball joint is pivotal in aircraft duct systems due to its favorable properties, including displacement compensation and flexibility. In the stress assessment of air ducts, ball joints are usually simplified by using “Joints” connections to reduce the convergence problems caused by non-linearity, which requires a high degree of accuracy in the characteristic parameters of the ball joint. Accordingly, this paper builds a high temperature and pressure fatigue test platform to investigate the bending characteristics of the ball joint at different temperatures and pressures and points out the limitations of the current method. Then, a method combining finite element analysis (FEA) and the BP neural network is proposed to obtain the characteristic parameters of the ball joint. The results showed that the bending process of the ball joint tended to have two typically different stiffness properties, which were high rigidity and low rigidity. The bending characteristics were strongly influenced by pressure, but less influenced by temperature. The existing test platform increased the force reaction at the contact areas of the ball joint, resulting in errors in the measurement of characteristic parameters. The BP neural network prediction method could effectively alter the ball joint properties and reduce errors.

## 1. Introduction

The air duct system, as the lifeline of the aircraft, is a vital part of the air management system. Its safety and reliability affect the function of the whole air management system. An unreliable air duct system can even pose potential safety hazards to other surrounding systems because the air ducts are distributed in multiple areas, such as the wing, engine suspension and fuselage [1,2,3,4,5]. Furthermore, the high temperature and pressure in the air ducts cause periodic mechanical stretching, compression and thermal fluctuations during the flight of the aircraft, and are liable to cause fracture and failure. In 2007, Japan Airlines issued a statement saying that cracks had been found in the ducts of nine McDonnell Douglas 90 (MD90) aircrafts, one of which made an emergency landing. The United States found cracks in the main engines of all four space shuttles in service, and for safety reasons, NASA decided to temporarily ground all of its space shuttles until the problem was clarified. Sujata et al. [6] described a twin-engine fighter accident caused by the fatigue failure of a fuel pipeline. All of these incidents were related to thermal fatigue and damage to the duct. Therefore, the design of air duct stress compensation has become one of the key technologies for aircraft air management systems [7,8]. Air ducts usually use compensators to solve the stress concentration problem. Ball joints are novel metal encapsulated compensators with strong displacement compensation ability and excellent high temperature and pressure durability, and are greatly favored by the aerospace industry in extremely harsh operating environments.

Recently, some researchers have carried out a series of studies on the behavior of the compensator. Gan et al. [9] found that the stress and displacement of the bellows were linearly distributed with the bending angle and that the stress of the inner bellows was smaller compared with the outer bellows. Ma et al. [10] used the FE method to analyze the performance of bellows and obtained the stress, frequency and vibration type under 0~1.2 MPa. Prasanna et al. [11] studied the effects of wall thickness, corrugation and convolution radius on the static mechanical characteristics of circular-type bellows. Yuan et al. [12] put forward a theoretical analysis of reinforced S-shaped bellows and discussed the mechanical behavior of bellows under different loads. Huo et al. [13] studied the bending performance of reinforced S-shaped bellows under a pressured load by a mathematical method and numerical simulation analysis. Jiang et al. [14] presented fittability to appraise the convolution shape precision of bellows and investigated the effect of different load types on the wall thickness and fittability of bellows. Xiang et al. [15] explored the influence of different loads on different convolution joints and obtained several characteristic curves. In addition, scholars have conducted a lot of research on the forming characteristics [16,17,18] and fatigue life [19,20,21] of bellows.

From the above studies, it can be concluded that many researchers have focused their attention on the effects of external loads and forming parameters, etc., on the performance of bellows. In addition, bellows have a high degree of freedom and are prone to damaging themselves and other components due to excessive duct displacement deformation when used in air ducts. In contrast to bellows, as a restrictive compensator, the ball joint limits the axial and torsional degrees of freedom. It offers displacement compensation and flexibility characteristics with enhanced reliability in air ducts. However, little research has been carried out on the characteristics of ball joints. Based on the differences in the properties of ball joints and bellows, the performance of bellows cannot fully reflect the characteristics of ball joints. Therefore, the results of the bellows analysis cannot be directly applied to the study of ball joint characteristics.

Ball joints have strong non-linear characteristics. In duct stress assessments, a large number of solid ball joint models tend to cause slow or even non-convergent nonlinear iterations [22]. To improve the convergence of the iterative process, a “Joints” connection is often used to simplify the solid ball joint. However, this requires the exact characteristic parameters of the ball joint, including the bending stiffness and bending friction torque. The bending characteristic parameters of the ball joint are closely related to the material of the ball joint, the fluid temperature and pressure in the duct, and also to the installation form of the ball joint [23]. Therefore, it is essential to study the compensation characteristics of the ball joint under full envelope flight conditions of the duct system, especially under extreme environmental conditions.

In this paper, a high temperature and pressure fatigue test platform is established to evaluate the bending characteristics of the ball joint. The angle-bending moment curves at different temperatures and pressures are investigated experimentally. The differences between the experimental procedure and the actual flight of the ball joint are considered and discussed. Finally, the parameters of the ball joint compensation characteristics that meet the requirements of practical use are predicted by combining numerical simulations and neural network methods.

## 2. Structural Composition and the Compensation Principle of Ball Joints

The ball joint, which is used for thermal compensation and to increase the flexibility of the air duct, is a non-detachable duct connection. The following factors should be considered when using the ball joint: (1) the fatigue strength of structures; (2) not exceeding the angle compensation limit; (3) leakage. The overuse of the ball joint is likely to cause excessive flexibility of the duct system and induce vibration. Therefore, it is necessary to study the characteristics of the ball joint for its application in air ducts.

The ball joint, also known as an angular bellows compensator, is a kind of restricted bellow. It relies on the expansion and contraction of bellows to release primary/secondary stress. Different from bellows, the ball joint has a limiting structure to avoid the excessive expansion and contraction of bellows. The sectional view of the ball joint is presented in Figure 1a. The structure is shown in Figure 1b. The ball joint is mainly composed of external support, an external ball twist, an internal ball twist, a deflector, and bellows. The external support, external ball twist, deflector and bellows are connected by welding, and the external ball twist and internal ball twist are connected by sliding friction. The wave number of bellows varies according to the size of the ball joint. Bellows are designed in a U shape and the thickness is 0.4 mm. However, the bellows will be deformed from a U to a Ω shape in the production process due to the need for structural coordination. This change is irregular and random. The bending is realized by the expansion and contraction of the bellows, and the maximum angle of bending is limited by the friction between the external and internal ball twist.

The ball joint is used as a stress compensator in the air duct to alleviate the stress concentration of the duct system. From a qualitative point of view, there is a bending friction torque of the ball joint. If the bending moment of the joint is greater than the bending friction torque, the joint begins to bend. The angle of bending has a certain linear relationship with the bending moment, as shown in Figure 2. The bending friction torque and bending stiffness are important parameters for evaluating the bending characteristics of the ball joint. The degrees of freedom of the ball joint are listed in Table 1. The ball joint has no axial displacement degrees of freedom, but only rotational degrees of freedom.

## 3. Experiment

### 3.1. Test Platform

A high-temperature and -pressure fatigue test platform was built to capture the bending characteristics of the ball joint. The experiment was completed on an electro-hydraulic servo fatigue testing machine, as presented in Figure 3. Figure 4 is a sketch of the experiment devices. The size of the ball joint was 2.5 inches. Straight ducts were welded at both ends of the ball joint to facilitate the installation of the specimen. One of the straight ducts was fixed on the support and the other was in a linear bearing which moved in a vertical direction and allowed the duct to slide appropriately within. The displacement load of the linear bearing was controlled by a fatigue testing machine. The ball joint was wrapped with the heating furnace. The ends of the straight ducts were blocked, and the pressure in the duct was guaranteed through the high-pressure gas cylinder.

The material of the specimen was GH4169, with a density of 8240 kg/m*^3^*, a tensile strength of 965 MPa and a yield strength of 550 MPa. The material properties of GH4169 at different temperatures are listed in Table 2.

### 3.2. Experimental Content

Temperature and pressure are common loads on the ball joint which have the greatest effect on the compensation characteristics of ball joints. The main mechanism of temperature is to change the properties of materials. In addition, the rigid state of each part of the ball joint, such as the mechanical characteristics of the bellows, will be changed under different pressures. During the actual flight, the aircraft will encounter different external conditions, and the control system will execute different control procedures. There will be great differences in the flow, temperature and pressure in the duct under the whole flight envelope. Therefore, capturing the motion and bending characteristics of the ball joint under different airflow parameters is important for the thermal compensation design of the air duct.

In this paper, the bending friction torque and bending stiffness characteristics of the ball joint under different temperatures (200 °C, 400 °C and 600 °C) and pressures (1 MPa, 1.5 MPa and 2 MPa) were investigated. The bending friction torque is usually expressed as M = FL, where F is the vector force and L is the frictional torque. It is defined as the value of the bending moment when the angle-bending moment curve of the ball joint enters “low rigidity”. The bending stiffness is the bending moment per unit angle under “low rigidity”.

### 3.3. Experimental Results

The angle-bending moment curves of the ball joint under different temperatures and pressures are shown in Figure 5. The bending process of the ball joint tended to have different mechanical behaviors. The bending moment had a linear relationship with the angle of bending before reaching the bending friction torque and the ball joint had high rigidity. After reaching the bending friction torque, the bending of the ball joint became easier. The angle of bending increased linearly with the bending moment, but bending stiffness was significantly lower than what it was previously. The characteristic parameters of the ball joint under different loads are listed in Table 3.

When the temperature was constant, the bending friction torque increased significantly with the increase in pressure, while the change in bending stiffness was small. Figure 6 shows the stress distribution nephogram of the internal ball twist and bellows. Under low pressure, the contact stress between the internal and external ball twist was small. The internal stress level of the bellows was low, and the reaction force on the other structures of the ball joint was small. With the increase in pressure, the ball twist fitted more tightly and the structural rigidity increased, resulting in the increase in the bending friction torque and bending stiffness. When the pressure was constant, the bending friction torque and bending stiffness decreased with the increase in temperature, but the decrease was limited. This was because the change in temperature led to the decrease in Young’s modulus and the increase in the coefficient of linear expansion, which increased the deformation of the specimen.

When the temperature was changed from 200 °C to 600 °C, the bending friction torque under different pressures was changed by 5.44%, 5.61% and 5.83%, and the bending stiffness was changed by 16.25%, 18.54% and 23.97%. When the pressure was changed from 1 MPa to 2 MPa, the bending friction torque under different temperatures was changed by 83.51%, 82.15% and 82.74%, and the changes in bending stiffness were 61.67%, 53.10% and 46.77%. Therefore, the bending characteristics of the ball joint were more strongly affected by pressure.

### 3.4. Applicability Analysis of Experimental Results

A force analysis of the ball joint in the experiment was further carried out. Both ends of the ball joint needed to be blocked in order to meet the maintenance of internal high temperature and high pressure. The pressure acting on the blockage surface was directly transmitted to the frictional contact surfaces of the internal and external ball twist, resulting in a significant increase in the force reaction of the contact surface, as shown in Figure 7. The increment was *F* = *PA*, where *F* is the vector force, *P* is the pressure, and *A* is the blockage area. The measured characteristic parameters were larger than the onboard conditions. Therefore, the measurement method of bending characteristics had poor adaptability and great limitations which needed to be corrected.

## 4. Correction Method for Bending Characteristics

### 4.1. Correction Method Introduction

The correction method for bending characteristics was proposed in this paper as shown in Figure 8. In order to correctly capture the bending characteristics of the ball joint under the actual onboard conditions, the experiment and FE analysis of the ball joint with both ends blocked were first completed. Secondly, the relative error of the data obtained from the experiment results and the FE results were compared and analyzed. At the same time, the FE analysis without the ends blocked was completed. Then, the BP artificial network model was built with the angle of bending, temperature and pressure as the input layers and the relative error as the output layer. Finally, the FE results without the ends blocked were corrected with the results were predicted by the BP neural network. The bending characteristics of the ball joint without the end blocked were obtained.

### 4.2. FE Analysis of a Ball Joint

An equal-scale model was established in UG. In order to simplify the model, the linear bearing was not modeled, and the loads were applied to the blocked end. The FE meshed model is shown in Figure 9.

There was one pair of frictional contact and five pairs of non-slip contact among the different structural contact relationships of the ball joint. A “Frictional Connection” was established between the frictional contact surface and the target surface to simulate the frictional contact relationship, and the friction coefficient was set to 0.8. The remaining face-to-face contact was set to “Bonded Connection” to simulate no-slip, no-separation contact. The contact algorithm adopted the augmented Lagrange multiplier method.

One end of the model was fixed, and the other end was applied by bending loads. The angle-bending moment curves were obtained under different loads. Pressure loads were applied to the internal walls of the duct and ball joint, and temperature loads were applied to the overall model. Material elastoplasticity, structural geometric non-linearity and contact non-linearity were fully considered in the FE analysis.

The bending characteristics of the ball joint with blocked ends were calculated using the FE method. The relative error of experimental results and FE results are shown in Figure 10. There was a close trend consistency between the experimental results and the FE results. The maximum value of the relative error occurred near the bending friction torque. However, different from the experiment results, the curves of the FE results were relatively smooth. There was no significant turning point (the point of bending friction torque), and there was a large error in the bending stiffness. The reason for this was that the change in the friction coefficient of the friction pair under different load conditions was not considered in the FE analysis. In addition, the bellows of the ball joint had undergone a certain extrusion deformation during the production process as shown in Figure 1a, and this change could not be reflected in the simulation process.

The bending characteristics of the ball joint without blocked ends were calculated, as shown in Figure 11. It could be found that when the end of the air duct was not blocked, the bending friction torque and bending stiffness of the ball joint were significantly reduced. The FE results could not perfectly describe the results under the onboard conditions. Therefore, it was necessary to correct the FE characteristic curves of the end without blockage.

### 4.3. Establishment of the BP ANN Model

The BP ANN model was established using Matlab. The relative errors of the experiment results and the FE results under different working conditions were studied. The input layer had three nodes, which were angle of bending, pressure and temperature. The hidden layer was a single hidden layer structure. The number of hidden layer nodes was selected to be 12. The output layer had one node, which was the relative error. The established network structure was 3 × 12 × 1.

Among nine groups of error angle moment curves, 450 data points were selected to establish a network, 80% of which were randomly selected for network training, and the remaining data points were used as test data to test the prediction ability of the BP neural network model.

The input data were normalized, and the output data were denormalized to make the input value between −1 and 1. The input and output data were normalized and denormalized according to Equations (1) and (2), as follows:

(1) $$X_{n}=\left(X-X_{\min}\right)/\left(X_{\max}-X_{\min}\right)$$

(2) $$X=X_{\min}+X_{n}\left(X_{\max}-X_{\min}\right)$$

where *X* is the primary data; *X*_min_ is the minimum value of specimen data; *X*_max_ is the maximum value of specimen data; and *X*_n_ is the normalized data.

The accuracy of the BP neural network model was systematically evaluated with correlation coefficient (R) and mean absolute relative error (AARE). R reflected the linear correlation strength between the expected value and the predicted value; AARE was used to verify the predictability of the model. R and AARE were defined as follows:

(3) $$R=\frac{\sum_{i=1}^{N}\left(X_{i}-\bar{X}\right)(Y_{i}-\bar{Y})}{\sqrt{\sum_{i=1}^{N}{\left(X_{i}-\bar{X}\right)}^{2}}\sqrt{\sum_{i=1}^{N}{\left(Y_{i}-\bar{Y}\right)}^{2}}}$$

(4) $$\mathrm{AARE}=\frac{\sum_{i=1}^{N}\left|\left(X_{i}-Y_{i}\right)/X_{i}\right|}{N}\times 100\%$$

where *X_i_* and *Y_i_* are the expected value and the predicted value, respectively; $\bar{X}\;$and $\bar{Y}\;$are the averages of *X_i_* and *Y_i_*, respectively; and *N* is the number of data points used for fitting.

After repeated training, tansig and purelin were selected as the activation functions. Trainlm was selected as the training function and the learning rate was 0.3. When the target of training error was set to 10^−5^ and the number of steps was set to 1000, the network had high accuracy.

The difference between the predicted value and the expected value was small, as shown in Figure 12. R and AARE were 0.9927 and 0.0051%. This could be used to predict the bending characteristics of the ball joint.

## 5. Results and Analysis

According to the FE results, it was found that when the ends were not blocked, the angle of bending corresponding to the bending friction torque decreased by 0.4~0.8°. However, the relative error curves predicted by the BP neural network would not shift. The obtained results of when the BP neural network was directly used to predict the bending characteristics are shown in the blue curve in Figure 13. The curve exhibited linear characteristics at 0~0.2°, and with the increase in the angle of bending, it tended to be non-linear. When the angle of bending exceeded 3°, it entered the linear stage again. The tendency of the non-linear stage was inconsistent with the actual law. Therefore, linear curves of 0~0.2° and 3~5° were selected to correct the bending characteristics of the ball joint under the onboard conditions. Then, the correction results were obtained by extending and intersecting the two lines. The final correction curve is the black curve in Figure 13. The bending properties under different loads were corrected, as shown in Figure 14.

Bending characteristics are listed in Table 4. The rigidity of the actual ball joint was much smaller than that under the condition of blocked ends. When the pressure was 2 MPa and the temperature was 200 °C, the bending friction torque was reduced by 77.91% compared with the blocked condition. The proposed method could effectively correct the characteristic parameters of the ball joint under actual onboard conditions.

## 6. Conclusions

The ball joint is a new structure to solve the stress concentration problem in air ducts. To obtain the bending characteristics of the ball joint at different temperatures and pressures, a test platform was constructed in this paper. Due to the limitations of the experimental measurements, the ball joint in the test platform was subjected to an additional force reaction that should not exist, making the measured data inaccurate. Therefore, this paper proposed a method combining FE analysis and the BP neural network to master the bending characteristics of the ball joint, which effectively solved the problem of the lack of additional force reaction at the end of the ball joint during its use. The following conclusions can be drawn:

(1) The bending process of the ball joint had two different stiffness characteristics, including high rigidity and low rigidity. Before reaching the bending friction torque, the bending moment was linearly related to the angle of bending, and the ball joint had high rigidity. After reaching the bending friction torque, the bending moment still increased linearly with the angle of bending, but the bending stiffness was significantly lower than before. The bending friction torque and the bending stiffness of the ball joint increased with pressure and temperature loading. However, the characteristic parameters of the ball joint were more sensitive to pressure;
(2) The bending characteristics of the ball joint with pressure blockages at both ends were studied by experimental and FE methods, respectively. The trends of the experimental and FE results were in some agreement. However, the curves of the FE results were relatively smooth and there was no obvious turning point (bending friction torque point). The reason for this was that the variation in the friction coefficient of the friction pair under different load conditions was not taken into account in the FE analysis;
(3) Due to the pressure blockage at both ends of the duct under experimental conditions, the force reaction at the contact area of the ball joint increased compared with the onboard conditions, leading to a large error in the traditional method of measuring the characteristics. Accordingly, a correction method for the bending characteristics of the ball joint combining FE analysis and the BP neural network was proposed. The method ensured that the “Joints” connections and the ball joint solids had the same stiffness characteristics, thus reducing the non-linearity caused by the simultaneous presence of multiple ball joint solids and speeding up the calculation and convergence during the stress assessment of the air ducts.

## Figures and Tables

**Figure 1:**
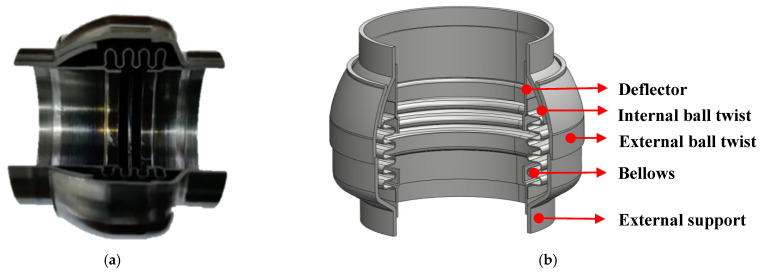
Schematic illustration of a ball joint: (**a**) sectional view; (**b**) structure of the ball joint.

**Figure 2:**
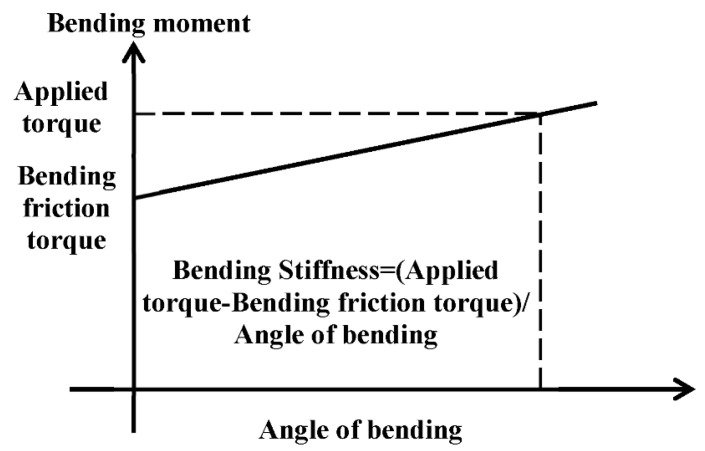
Ideal bending characteristics of a ball joint.

**Figure 3:**
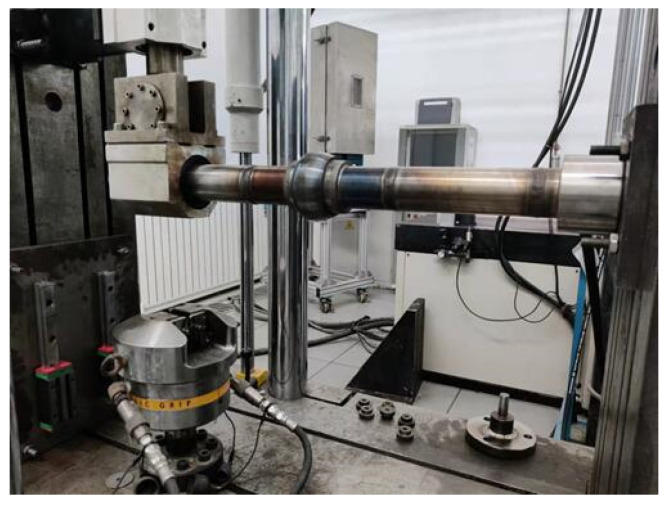
Test platform.

**Figure 4:**
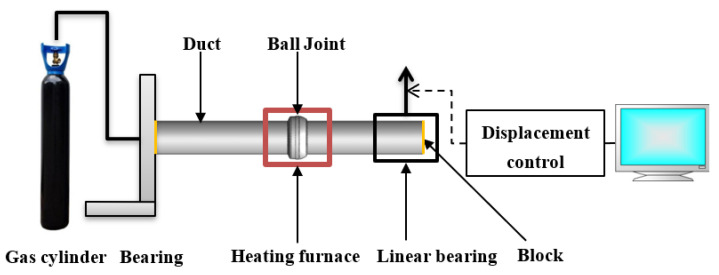
Schematic diagram of the experiment.

**Figure 5:**
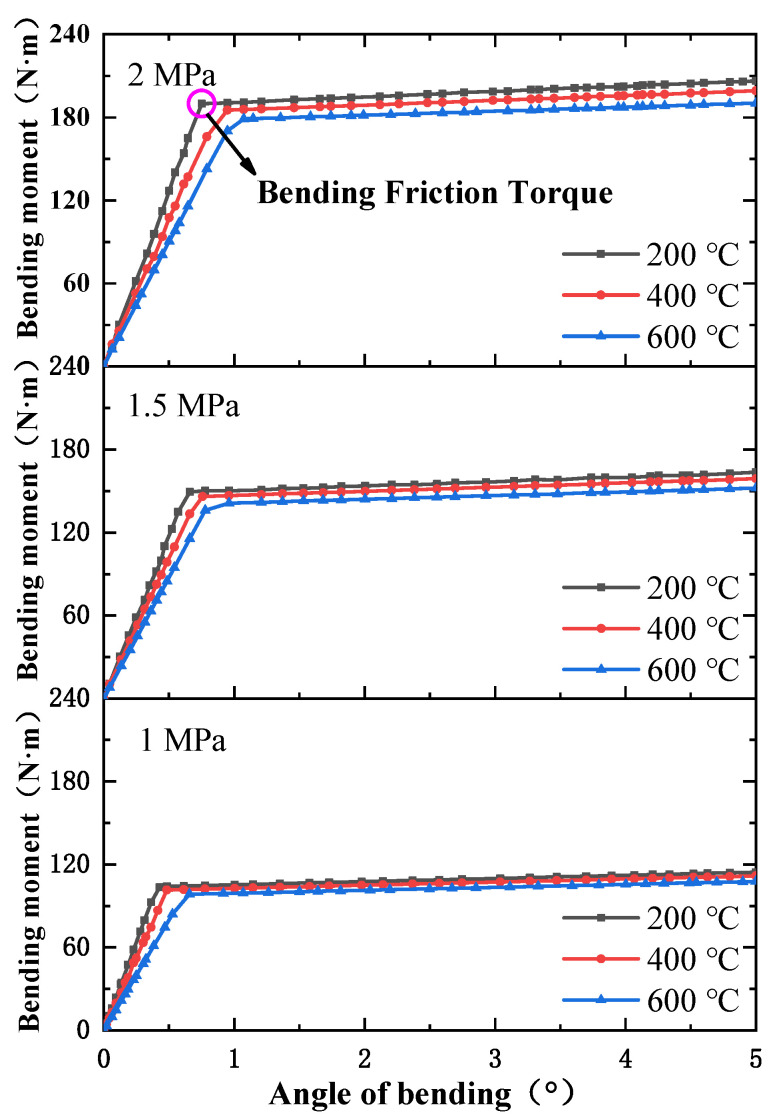
Bending characteristic curves of ball joints under different loads.

**Figure 6:**
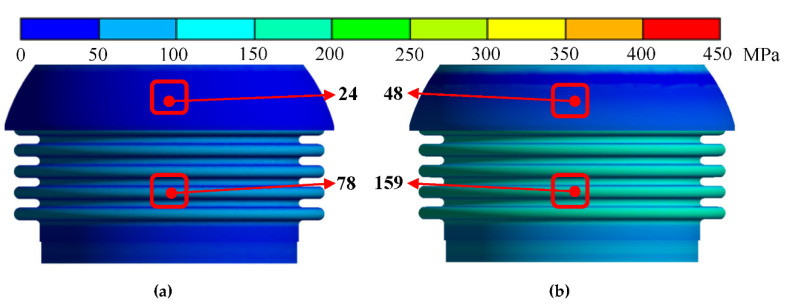
Stress nephogram under different pressures (T = 200 °C): (**a**) 1 MPa; (**b**) 2 MPa.

**Figure 7:**
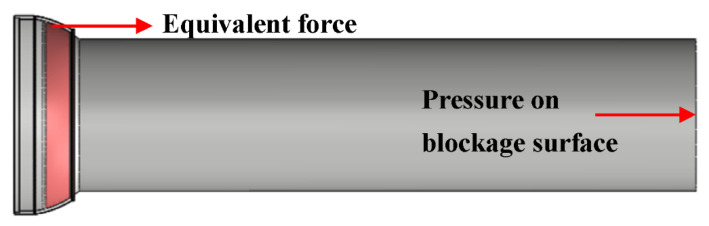
Force analysis.

**Figure 8:**
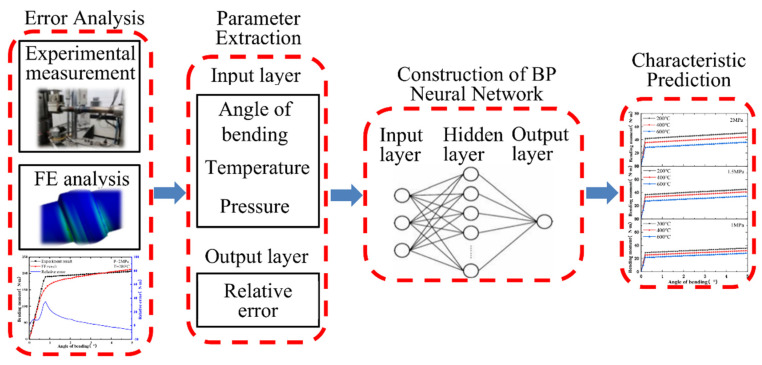
Correction method based on the BP neural network.

**Figure 9:**
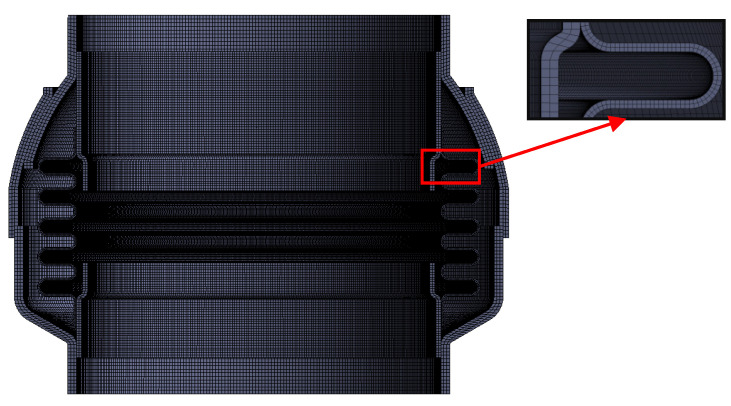
FE meshed model.

**Figure 10:**
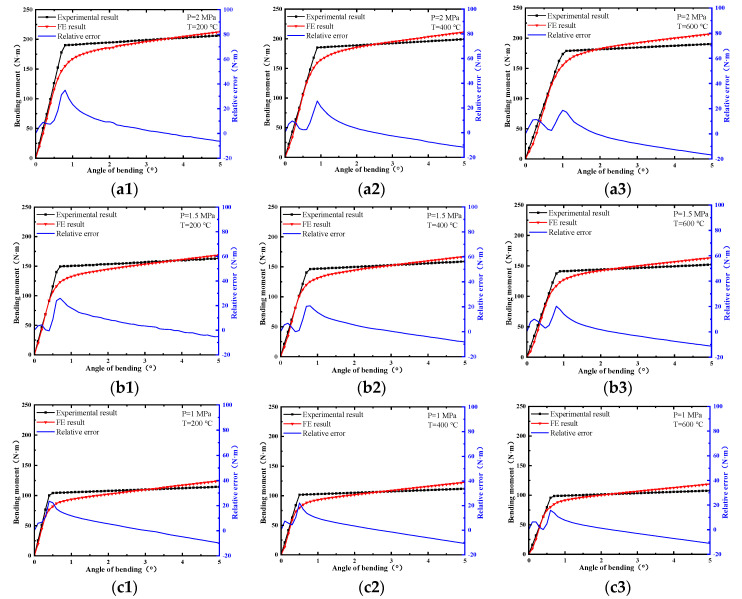
The relative error of experiment results and FE results: (**a1**) P = 2 MPa, T=200 °C; (**a2**) P = 2 MPa, T = 400 °C; (**a3**) P=2 MPa, T=600 °C; (**b1**) P=1.5 MPa, T = 200 °C; (**b2**) P=1.5 MPa, T = 400 °C; (b3) P = 1.5 MPa, T = 600 °C; (**c1**) P=1 MPa, T = 200 °C; (**c2**) P=1 MPa, T = 400 °C; (**c3**) P=1 MPa, T = 600 °C.

**Figure 11:**
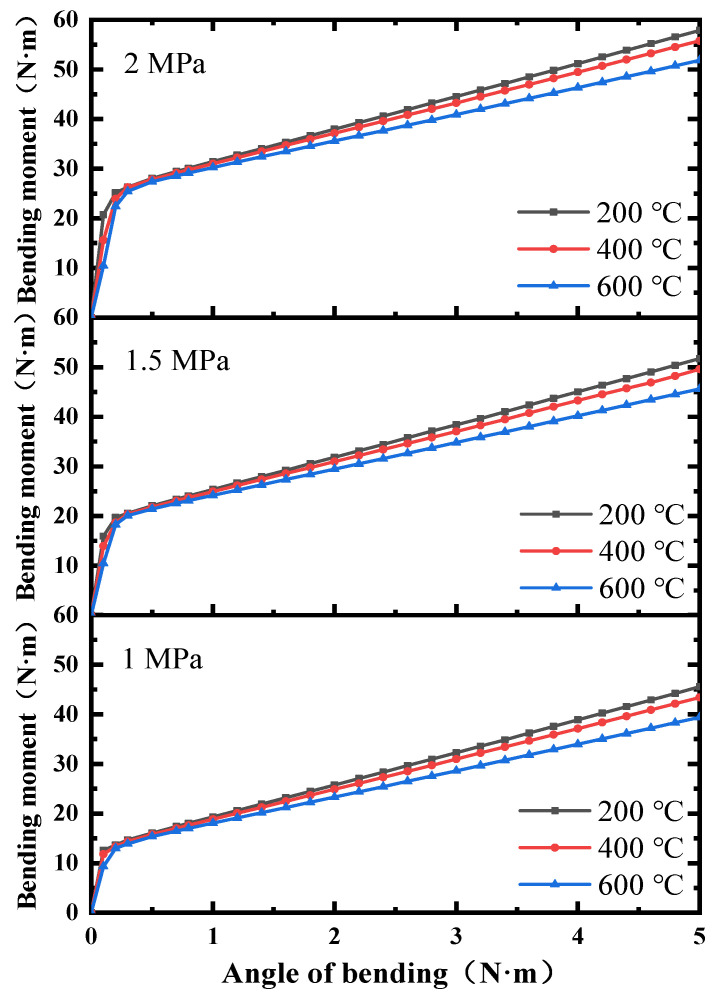
Bending characteristics of the ball joint without the ends blocked.

**Figure 12:**
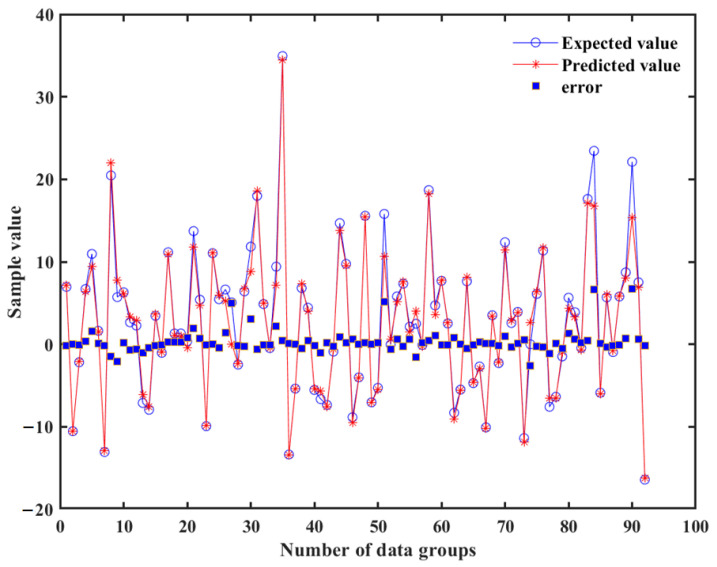
Comparison of predicted values and expected values of the BP neural network test set.

**Figure 13:**
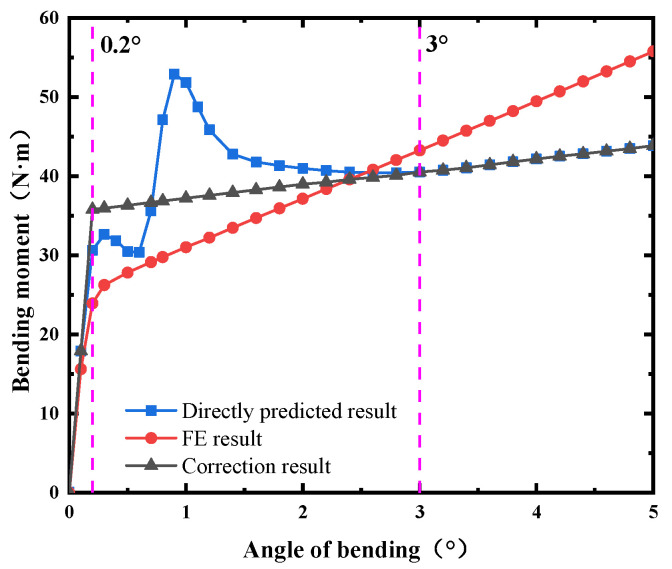
Method introduction.

**Figure 14:**
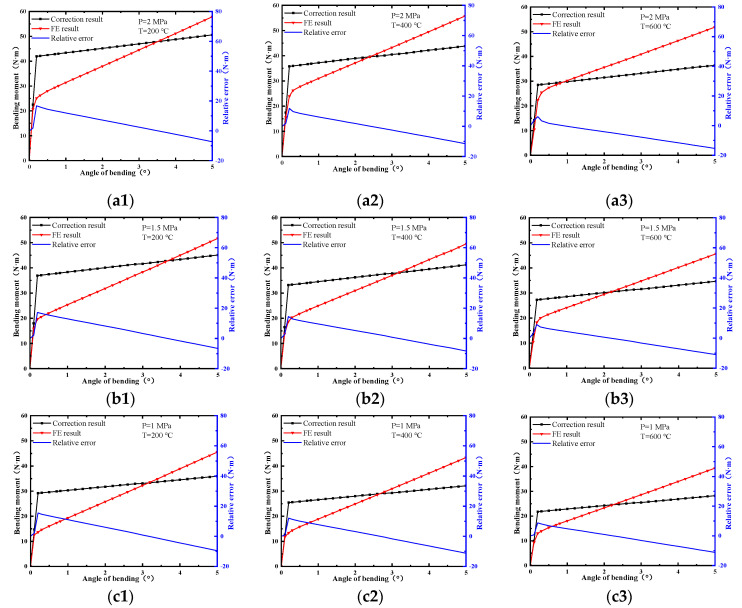
Corrected bending characteristics: (**a1**) P = 2 MPa, T = 200 °C; (**a2**) P = 2 MPa, T = 400 °C; (**a3**) P = 2 MPa, T = 600 °C; (**b1**) P = 1.5 MPa, T=200 °C; (**b2**) P = 1.5 MPa, T = 400 °C; (**b3**) P = 1.5 MPa, T = 600 °C; (**c1**) P = 1 MPa, T = 200 °C; (**c2**) P = 1 MPa, T = 400 °C; (**c3**) P=1 MPa, T = 600 °C.

**Table 1:** Degrees of freedom of a ball joint in different directions.

Maximum Angle of Bending (°)	Torsional Stiffness (N·m/°)	TX	TY	TZ	Rot RX	Rot RY	Rot RZ
±5	Rigid	Rigid	Rigid	Rigid	Rigid	curve	curve

**Table 2:** Material properties of GH4169 at different temperatures.

Temperature (°C)	Young’s Modulus (GPa)	Coefficient of Linear Expansion (10^−6^C^−1^)	Poisson’s Ratio
20	204	11.8	0.3
200	189	13.0	0.3
400	176	14.1	0.3
600	150	14.8	0.32

**Table 3:** Characteristic parameters of ball joint under different loads.

	Bending Friction Torque (N·m)			Bending Stiffness (N·m/°)		
	1 MPa	1.5 MPa	2 MPa	1 MPa	1.5 MPa	2 MPa
200 °C	103.50	149.35	189.93	2.40	3.29	3.88
400 °C	101.57	146.11	185.01	2.26	3.02	3.46
600 °C	97.87	140.97	178.85	2.01	2.68	2.95

**Table 4:** Correction value of bending characteristics.

	Bending Friction Torque (N·m)			Bending Stiffness (N·m/°)		
	1 MPa	1.5 MPa	2 MPa	1 MPa	1.5 MPa	2 MPa
200 °C	29.16	36.93	41.95	1.45	1.68	1.8
400 °C	25.26	32.98	35.79	1.43	1.65	1.76
600 °C	21.76	27.29	28.46	1.37	1.58	1.67

## Data Availability

Not applicable.
